# Closing the mental health treatment gap in South Africa: a review of costs and cost-effectiveness

**DOI:** 10.3402/gha.v7.23431

**Published:** 2014-05-15

**Authors:** Helen Jack, Ryan G. Wagner, Inge Petersen, Rita Thom, Charles R. Newton, Alan Stein, Kathleen Kahn, Stephen Tollman, Karen J. Hofman

**Affiliations:** 1 Department of Psychiatry, University of Oxford, Oxford, UK; 2 MRC/Wits Rural Public Health and Health Transitions Research Unit (Agincourt), School of Public Health, Faculty of Health Sciences, University of the Witwatersrand, Johannesburg, South Africa; 3 Umeå Centre for Global Health Research, Umeå University, Umeå, Sweden; 4 School of Applied Human Sciences, University of KwaZulu Natal, Durban, South Africa; 5 PRICELESS SA (Priority Cost Effective Lessons in System Strengthening South Africa), School of Public Health, Faculty of Health Sciences, University of the Witwatersrand, Johannesburg, South Africa; 6 INDEPTH Network, Accra, Ghana

**Keywords:** mental health, South Africa, economics, health planning, policy, costs and cost analysis

## Abstract

**Background:**

Nearly one in three South Africans will suffer from a mental disorder in his or her lifetime, a higher prevalence than many low- and middle-income countries. Understanding the economic costs and consequences of prevention and packages of care is essential, particularly as South Africa considers scaling-up mental health services and works towards universal health coverage. Economic evaluations can inform how priorities are set in system or spending changes.

**Objective:**

To identify and review research from South Africa and sub-Saharan Africa on the direct and indirect costs of mental, neurological, and substance use (MNS) disorders and the cost-effectiveness of treatment interventions.

**Design:**

Narrative overview methodology.

**Results and conclusions:**

Reviewed studies indicate that integrating mental health care into existing health systems may be the most effective and cost-efficient approach to increase access to mental health services in South Africa. Integration would also direct treatment, prevention, and screening to people with HIV and other chronic health conditions who are at high risk for mental disorders. We identify four major knowledge gaps: 1) accurate and thorough assessment of the health burdens of MNS disorders, 2) design and assessment of interventions that integrate mental health screening and treatment into existing health systems, 3) information on the use and costs of traditional medicines, and 4) cost-effectiveness evaluation of a range of specific interventions or packages of interventions that are tailored to the national context.

Mental, neurological, and substance use (MNS) disorders accounted for 10% of the global burden of disease (GBD) in 2010 (1), yet on average, mental health accounts for less than 1% of national health budgets in Africa and South East Asia (2). In South Africa, as in many low- or middle-income countries (LMICs), the burden of mental disorders has grown over the past 20 years (1990–2010) (1). This rise is expected to continue, in part due to the ongoing epidemiological transition from communicable to non-communicable diseases (NCDs) (3) and co-morbidity between MNS disorders, HIV, and other chronic health conditions (4–6).

In 2011, faced with this growing burden, South Africa's Ministry of Health publically committed to increasing by 30% the number of people screened and treated for mental disorders by 2030, and to reducing by 20% per capita alcohol consumption by 2020 (5, 7, 8). Health budgets and system designs, however, do not currently reflect these new commitments. For example, the District Specialist Teams introduced under the proposed national health insurance provide key specialty services to supplement primary health care at the district-level, but the teams do not include mental health providers (9). Better understanding of cost-effective, context-specific interventions or packages of interventions for treatment and prevention of MNS disorders may contribute to achieving South Africa's ambitious mental health targets.

Economic data is one of several relevant factors, including burden of disease and equity, that policymakers and donors consider as they set priorities and make spending choices in resource-limited settings. Economic data provides an ‘external frame’ that can be used to make comparisons between competing priorities or interventions and justify investment in programs (10, 11). Cost-effectiveness analyses (CEAs), which compare interventions to determine those likely to yield the most improvements in health per dollar (12), have achieved notable successes as an advocacy tool for system improvements (13, 14).

Despite the importance of economic information in advocating for and designing policy change, there are no reviews of costs related to MNS disorders and cost-effectiveness of their treatment in South Africa, and few reviews that examine costing aspects of MNS disorders in any LMICs (2). Compiling the disparate information on costs and cost-effectiveness at a country-level could facilitate identification of key needs, interventions, and knowledge gaps. A health system approach, which goes beyond single disorders or interventions, is necessary because MNS disorders often occur together and may be co-morbid with other health conditions, and a holistic view can inform the design of treatment packages (15).

Mental health care in South Africa differs from that in other LMICs, making a South Africa-specific review necessary. Although South Africa's gross national income (GNI) bears greater similarity to other middle-income countries than to the rest of sub-Saharan Africa, findings on MNS disorders from middle-income nations, including those in Latin American or South East Asia, cannot be generalized to South Africa. South Africa has a unique post-*apartheid* socioeconomic and cultural context of inequality, with one of the highest Gini coefficients globally (a statistical measure of income inequality in a population) (16), and particular disparities between rural and urban areas. It also has a complex disease burden characterized by high HIV prevalence and a growing burden of non-communicable chronic conditions (17).

Given the paucity of economic assessments of mental health in sub-Saharan Africa and the specific challenges facing South Africa's mental health system, this review summarizes current understanding and highlights key knowledge gaps. Findings may inform future research and the design of mental health policy and interventions in South Africa, other nations in sub-Saharan Africa, and settings with a high prevalence of conditions, including HIV, that may be co-morbid with mental disorders.

## Prevalence and epidemiological burden of MNS disorders in South Africa

Globally, the World Health Organization (WHO) estimates that 30.8% of all years lived with disability (YLDs) are due to neuropsychiatric disorders, primarily unipolar depression (11.9%), alcohol use disorder (3.1%), schizophrenia (4.8%), and bipolar mood disorder (4.4%) (18). Three MNS disorders (unipolar depressive disorders, self-inflicted injuries, and alcohol use disorders) are among the top 20 causes of disability-adjusted life years (DALYs) lost globally (18, 19), and MNS disorders account for a larger percentage of lost DALYs than cardiovascular disease or cancer.

The South African Stress and Health (SASH) Study, conducted between 2002 and 2004, provides the only nationally representative data on the prevalence of common mental disorders (20, 21). Other prevalence studies examine specific populations and disorders (22, 23), but do not provide the national representativeness of the SASH (24). Table 1 shows that lifetime prevalence of common mental disorders was 30.3%, and prevalence in the 12 months prior to the survey was 16.5%.

**Table 1:** Prevalence of select categories of MNS disorders in South Africa and other LMICs countries included in the WHO World Mental Health Survey Initiative

MNS disorder	Lifetime prevalence (South Africa) (%)	12-month prevalence (South Africa) (%)	Lifetime prevalence (China) (%)	Lifetime prevalence (Columbia) (%)	Lifetime prevalence (Lebanon) (%)	Lifetime prevalence (Mexico) (%)	Lifetime prevalence (Nigeria) (%)	Lifetime prevalence (Ukraine) (%)
Anxiety disorders	15.8	8.1	4.8	25.3	16.7	14.3	6.5	10.9
Substance use disorders	13.3	5.8	4.9	9.6	2.2	7.8	3.7	15.0
Mood disorders	9.8	4.9	3.6	14.6	12.6	9.2	3.3	15.8
*Any disorder*	30.3	16.5	13.2	39.1	25.8	26.1	12.0	36.1

Sources: Williams et al. [20], Herman et al. [24], Kessler et al. [25].

SASH, part of the WHO World Mental Health Survey Initiative, is a cross-national effort to collect country-specific epidemiological data on mental disorders using a single assessment tool and data collection methodology. It includes seven LMICs: China, Columbia, Lebanon, Mexico, Nigeria, South Africa, and Ukraine. Lifetime prevalence rates of select types of MNS disorders are shown in Table 1, (25). Examining all countries surveyed, the highest lifetime prevalence of these disorders is in the US and New Zealand (47.4 and 39.3%, respectively) while the lowest is in Nigeria and China (12.0 and 13.2%, respectively). South Africa has more than twice the lifetime prevalence of mental and substance use disorders than Nigeria (12.0%), the only other African country surveyed (21, 26), and a greater lifetime prevalence than all LMICs except Columbia and Ukraine. However, these comparisons must be interpreted cautiously because researchers acknowledge that prevalence may be underreported in Nigeria and China due to stigma and lack of public familiarity with surveys (27); prevalence of impulse control disorders are measured in all LMICs except South Africa, potentially altering the overall prevalence estimate (25); and the manifestations of mental disorders and their diagnostic criteria may vary between cultural contexts, making any single diagnostic instrument potentially unfit to capture the range of ways mental disorders may be expressed (28). In this review, we have chosen to focus our epidemiological examination only on data from the WHO World Mental Health Survey to ensure uniform data collection methodology and consistency in definitions of mental disorders. Overall, there are less epidemiological data on mental disorders than on other disorders in LMICs and few panel data available to examine change in their prevalence or burden over time (29).

The SASH data suggest that the high prevalence of common mental disorders may be caused by exposure to stress and trauma during *apartheid* and the ongoing period of racial tension and inequality following *apartheid* (21). The SASH data shows that 74.8% of South Africans have experienced at least one traumatic event, most commonly trauma related to someone close to them (for example, death of a friend or family member), witnessing a traumatic event, or being the victim of criminal or intimate partner violence (20). These traumas and other life stressors, such as economic hardship and relationship problems, were associated with increased 12-month and lifetime prevalence of common mental disorders (30).

In 2000, neuropsychiatric disorders (including mental and nervous system disorders) ranked third in their contribution to South Africa's national burden of disease. Table 2 shows the contributions of individual MNS disorders to that burden (31). YLD data was not directly collected for the country and, consequently, DALY and YLD estimates must be interpreted cautiously. Suicide, the only MNS disorder in the top 20 leading causes of YLLs, ranked thirteenth (1.3%). Suicide causes 5,514–7,582 deaths per year (17), with the number of suicide deaths of people aged under 35 and over 65 increasing between 1968 and 1990 (32).

**Table 2:** Contributions of MNS disorders to South Africa's burden of disease

MNS disorder	Percentage of burden of disease (South Africa)	Ranking in contribution to burden of disease (South Africa)
Unipolar depressive disorders	5.8	2
Alcohol use	2.8	6
Bipolar mood disorder	2.1	9
Schizophrenia	2.1	11
Drug use	1.6	14
Foetal alcohol syndrome	1.1	16
Obsessive compulsive disorder	1.0	18
Panic disorder	1.0	19

Source: Norman et al. [31].

MNS disorders are commonly co-morbid with HIV, and the conditions are mutually reinforcing (6, 24, 33). Considerable research has focused on the high prevalence of common mental disorders in HIV-positive patients (33–36), with one study reporting that 35% of HIV patients in South Africa (n=100) meet the criteria for major depressive disorder, 6% for bipolar mood disorder, and 21% for generalized anxiety disorder (33), far higher than prevalence estimates for the general population (24, 33). Among patients with severe mental illness admitted to a psychiatric hospital (n=206), 29.1% were HIV positive, nearly triple the general population prevalence (37). These co-morbidity data may not reflect the current situation, as they were collected before the rollout of ARVs and at the tertiary health care level where people with the most advanced HIV-related disease present for treatment; those who did not go to the hospital or who visited primary care clinics were not included.

Depression and other mental disorders are of particular concern in patients with HIV because they can lead to suboptimal treatment adherence, and consequently, lower CD4 counts, increased viral load, and a greater chance of developing drug-resistant strains of HIV that require more costly second-line anti-retroviral therapy (38). A diagnosis of HIV also complicates treatment of MNS disorders because of interactions between antiretroviral drugs and other medications. Phenobarbital, for example, a common treatment for epilepsy in sub-Saharan Africa, substantially reduces the half-life of some anti-retrovirals, lowering their therapeutic efficacy (39).

There has been little published research on co-morbidities between MNS disorders and other chronic diseases, yet existing data from South Africa and elsewhere in sub-Saharan Africa suggests association between mental disorders and diabetes, stroke, and epilepsy (40–45).

## Service delivery infrastructure

In 2005, South Africa devoted 2.7% of its health budget to mental health care (19), more than twice that of Ghana, Uganda, and many other low- to middle-income countries (46), but less than high-income nations, such as the UK, which uses 10.8% of its health budget on mental health. Brazil and India, South Africa's middle-income peers, spend 2.38 and 0.06% of their health budgets on mental health, respectively (47). Mental health care budget data in the WHO's Mental Health Atlas was updated in 2011, but did not include new South African budget data. Consequently, 2005 data, the most recent available, is given here for South Africa.

As in many LMICs, the mental health system in South Africa is fragmented. Mental health care in South Africa has historically been reliant on psychiatric hospitals, with little attention to mental health in primary care (19, 48, 49). Currently, care for psychiatric disorders, epilepsy and other neurological disorders often occurs in silos, even at the same health facility, and also varies from urban to rural areas. For example, epilepsy may be treated by mental health care providers in rural areas, but by physical health care providers in urban areas. Substance use disorders are also treated in both the health sector and in social development. While the policy is to develop comprehensive care at a primary care level, this is not yet fully realized. Importantly, in South Africa, many people use traditional medicines for MNS disorders, often before or instead of seeking conventional medical treatment (50, 51).

With respect to human resources, there is a substantial mental health workforce shortage, with 1.2 psychiatrists and 7.5 psychiatric nurses per 100,000 people, nearly 10 times less than many high-income countries. South Africa's mental health professionals are concentrated in urban locations, with some rural provinces having one or no psychiatrist, leading to great disparities in care (19, 48).

### 

#### Review methods

For this narrative overview, we searched Google Scholar and MEDLINE (using PubMed and Ovid) for articles written in English on the economic burden of MNS disorders and for costing data on mental health interventions in South Africa and other sub-Saharan African countries. The narrative overview strategy was selected because it facilitates outlining an area of research that has previously not been widely discussed and highlights key theoretical or empirical gaps in the existing knowledge, yet does not fulfil the methodological criteria of a systematic review (52). To ensure that results provided a sufficient and broad overview of the existing knowledge, we used a deductive approach, generating the paper's headings (direct costs, indirect costs, CEAs) then searching for studies that fit those categories. We broadened the search criteria (from South Africa to sub-Saharan Africa) if there was little literature from South Africa, a strategy that would not be appropriate for a systematic review, but was called for in this case because the amount of economic data available from South Africa and whether data from elsewhere in sub-Saharan Africa is generalizable varies widely between different economic themes (i.e. direct costs, indirect costs, cost-effectiveness). We screened the search results for relevant, methodologically rigorous studies and conducted a forward search of the references of many of the relevant results to identify additional studies. Table 3 displays a summary of all of the studies included in the narrative overview.

**Table 3:** Summary of articles included in the narrative overview

Economic information available	Number of studies (total: 18)	Themes (number of study in parentheses)	Study setting (number of study in parentheses)
Direct costs	5	Private sector chronic care (2), public sector workforce costs (2), community interventions (1)	South Africa (5)
Indirect costs	4	Income loss from depression (1), severe mental disorders (1), hospital stay for mental disorder (1), and psychological distress (1)	Ghana (1), Kenya (1), Nigeria (1), and South Africa (1)
Cost-effectiveness	9	Cost-effectiveness of interventions for depression (1), epilepsy (1), bipolar mood disorder (1), heavy alcohol use (2), schizophrenia (1), and many mental disorders (2); cost-effectiveness of group psychotherapy (1)	Low- and middle- income regions, including sub-Saharan Africa (7), Nigeria (1), Uganda (1)

Language in South Africa's mental health policy focuses on mental health and substance use disorders (53), but we broadened the definition to MNS disorders to include patients with neurological disorders, such as epilepsy, who are often treated in mental health care facilities alongside those with mood disorders, anxiety, schizophrenia, and substance use disorders. Neurological and substance use disorders are also often co-morbid with mental disorders and share many of the same co-morbidities as mental disorders, such as HIV and other NCDs. Because the mental health system addresses MNS disorders and these disorders are interlinked, an economic analysis would not be complete without attention to the full burden on the system.

## Results

### Economic burden of MNS disorders

Economic costs due to mental illness are typically divided into direct and indirect costs (12, 54). Individuals, governments, health insurers or other institutions pay direct costs, usually the costs of medical care and services. Indirect costs include funds spent or lost as a result of the condition, including lost productivity for patients and caregivers, unemployment and disability benefits, and legal, penal, or other costs related to a crime. While distinguishing direct from indirect costs is useful, there is no defined reference case for measuring costs and classifying them as direct or indirect. As a result, there is considerable methodological variation between studies.

#### Direct costs

Two studies have explored the direct costs of private, outpatient chronic disease care in South Africa. One examined the costs of caring for patients who have private health insurance with chronic disease benefits and have been diagnosed with schizophrenia, epilepsy, or bipolar mood disorder (n=210,664 health insurance beneficiaries receiving treatment for at least one chronic condition—no specific data on number receiving treatment for other chronic mental disorders). In 2001, outpatient medical management (primarily tests, scans, and doctor visits) for 1 year ranged from R875 (USD$88) for an individual with bipolar mood disorder to R1200 (USD$120) for an outpatient with schizophrenia or epilepsy. Medication costs, on average, are much higher, ranging from R4362 (USD$436) for patients with epilepsy to R7287 (USD$729) for those with schizophrenia and R7512 (USD$751) for those with bipolar mood disorder (55). Additionally, based on data from a private sector pharmaceutical group, in 2008, prescriptions for Alzheimer's disease cost an average of R2659 (USD$266) per patient per year (n=588 patients) (56).

Public sector workforce expenditures in LMICs account for a substantial portion of health care costs (57), and likely a larger portion of mental health care costs because mental health services, particularly those with adequate capacity for psychosocial care, rely less on laboratory tests or tools and more on trained workers than other forms of healthcare (2). The estimated workforce cost of providing integrated adult mental health services for a limited number of priority mental disorders using a task-shifting approach (dedicating and supporting counsellors and community health workers to work in mental health rather than hiring more expensive specialist health mental workers) in primary health care in South Africa was £28,457 per 100,000 population (approximately USD$44,200 or USD$0.44 per person in the population served by the primary health care facility). The staffing costs of scaling-up integrated primary mental health care, and employing a task-shifting approach was cheaper than alternative staffing models to provide comparable care coverage, although the exact cost difference was not specified (58). The staffing costs associated with implementing inpatient and outpatient child and adolescent mental health care ranged from $5.99 per individual in the population to provide care for 15–30% of children and adolescents with mental disorders to $21.50 for care for 100% of children and adolescents with mental disorders (59). A randomized control trial in South Africa examined the effects of home-visits for recently discharged psychiatric patients (n=51) that aimed to prepare the family for home care and assessed health status and care over a 1-year period. This intervention reduced re-admission by 31.5% and the number of days spent in hospital in a year by 55.6%, producing a cost saving of R786 (approximately USD$79) per patient (60).

#### Indirect costs

Indirect costs, i.e. costs to families and households, may contribute to the total economic burden of mental illness more than direct costs (54). Only one study has assessed indirect costs in South Africa, but several have examined these costs elsewhere in sub-Saharan Africa.

In terms of productivity, the presence of severe depression or anxiety was associated with a reduction in personal income of USD$4798 per adult per year in South Africa, resulting in a national loss of USD$3.6 billion annually (61). In contrast, a Nigerian study found the annual impact of a severe mental disorder on productivity was USD$463 per patient, totalling USD$166.2 million annually (62). The disparity in these costs may be due to differences in purchasing power parity (PPP) between countries and different measurements of productivity. Examining the indirect costs of the institutional care system, a Kenyan study estimated lost productivity over the course of a hospital stay for patients and their families, showing that one psychiatric hospital admission resulted in a USD$453 productivity loss (63). Conversely, household survey data from Ghana examined the general population, many of whom lack access to mental health treatment, and showed that psychological distress, measured using the Kessler 10 Psychological Distress Scale (64), was associated with unemployment and lost work time in both formal and informal sectors. Individuals with moderate or severe psychological distress had reductions in productivity of 11.1 and 24.4%, respectively. From these estimates, the researchers calculated that psychological distress in Ghana is associated with an approximately 6.8% GDP loss, or USD$2.7 million annually (65).

Despite research indicating that common mental disorders co-occur with HIV in South Africa (35–37), no studies specify the direct or indirect costs that stem from the impact that MNS disorders have on other chronic conditions, including diabetes, and stroke.

### CEAs of mental health interventions

Existing research suggests that standard treatments, including psychotropic medications and various forms of psychotherapy, are effective in LMICs (66), and preliminary cost-effectiveness data suggests that they may also provide good value for money in terms of DALYs averted.

Several studies examine the cost-effectiveness of interventions for depression (67), epilepsy (68), bipolar mood disorder (69), heavy alcohol use (70, 71), and schizophrenia (72) in low-income regions, including sub-Saharan Africa. Using the same methodology, a single study compared the cost-effectiveness of interventions to address all of these disorders in sub-Saharan Africa. By estimating staffing, drug, and patient care costs (inpatient stay, laboratory tests, outpatient visits, medications), the researchers found that national or regional alcohol control policies (USD$117 per DALY averted by increasing taxation by 50%) and treatment for epilepsy or depression in primary care (epilepsy: USD$265 per DALY averted; depression: USD$858 per DALY averted using newer anti-depressants) were most cost-effective, while inpatient care for schizophrenia using newer psychotropic drugs (USD$11,072 per DALY averted) was least cost-effective. Treating schizophrenia (USD$2,748 per DALY averted) and bipolar affective disorder (USD$2,551 per DALY averted) in the community using older psychotropic drugs paired with psychosocial care was more cost-effective than inpatient treatment (schizophrenia: USD$6,816 and bipolar affective disorder: USD$4,874 per DALY averted). In general, treatments administered in community and primary care settings were more cost-effective than those in hospitals. There were substantial differences in cost-effectiveness between sub-Saharan African and South East Asian regions, underscoring the importance of context-specific cost-effectiveness data (73).

Within sub-Saharan Africa, the only country-specific cost-effective analysis for a range of interventions was conducted in Nigeria and found approximate correspondence with the regional data in the rank order of cost-effectiveness of interventions, but differences in the cost-effectiveness ratios for each intervention (26, 73). A CEA of group psychotherapy for individuals with depression in Uganda found that the therapeutic intervention cost $1,150 per quality-adjusted life year added. The authors concluded that this intervention was cost-effective because it cost less than Uganda's per capita GDP (74), the level that the WHO Commission on Macroeconomics and Health suggests as the upper limit for ‘highly cost-effective’ interventions (75). However, the study's authors acknowledged that that there is no universally recognized definition of ‘cost-effectiveness’ or criteria for what makes an intervention cost-effective in any given context.

## Discussion

This narrative overview examines available costing data on MNS disorders and the cost-effectiveness of treatments, with a focus on South Africa and data relevant to South Africa. In the public sector, there is data on workforce expenses at a population level for delivery of specific packages of care (55). In the private sector, there is information on the costs of medication and medical management for three severe and chronic mental disorders (58–60). Only one South African study investigated income reduction associated with depression and anxiety (61), and few other studies estimate indirect costs elsewhere in sub-Saharan Africa (62, 63, 65). There are some region-level data on the cost-effectiveness of interventions for the treatment and prevention of MNS disorders in sub-Saharan Africa, but no data specific to South Africa, even though existing analyses suggest variation in costs and cost-effectiveness between countries and regions.

### What we can conclude from what we know

The data in this review suggests that indirect costs from foregone income due to MNS disorders are substantial (61–63, 65), and there are cost-effective interventions for addressing them (73). The most cost-effective interventions incorporate mental health care into primary care or community services without the use of specialized workers (58–60, 73). Such integration may be particularly apt in South Africa because of the high and growing prevalence of MNS disorders co-morbid with HIV and likely, with other chronic conditions (3, 17, 35–37). Integrated interventions could improve coverage of a population that is at high risk for mental disorders and already presenting for care, which would maximize the impact and cost-effectiveness of interventions and improve overall health outcomes by increasing adherence to chronic disease treatment regimes.

Prevention interventions that address alcohol consumption by raising taxes, limiting advertising, or reducing alcohol availability by restricting hours of sale or increasing the drinking age have been shown to be highly cost-effective interventions for reducing DALYs lost due to MNS disorders in LMICs (71, 73). The low cost of these interventions suggests that other prevention programs, such as campaigns to reduce prevalence of risk factors for mental disorders (such as child abuse and sexual violence), may also prove cost-effective.

### Knowledge gaps

This review reveals four linked knowledge gaps and associated methodological challenges.

First, there is inadequate research on the health and financial burdens of MNS disorders in South Africa and other LMICs. In terms of health burden, much of the existing research examines prevalence; this likely underrepresents the burden of MNS disorders because of underreporting due to stigma and because much of the burden is due to disability and premature mortality from co-morbid conditions or poor lifestyle and self-care (6). For instance, individuals receiving public mental health services in the US died 13–30 years sooner than people in the general population, although the causes of death were similar to those of the general population (76). Premature mortality in people with mental disorders is not well understood in LMICs.

While DALY measurements, in theory, illustrate disability YLD and premature mortality (YLL) more effectively, consideration must be given to how the DALY is constructed and measured. DALY estimations in South Africa use disability weights that are not context-specific. Disability weighting for YLD calculations varies widely by context depending on the impact of a given condition on a person's lifestyle. In order to develop more accurate burden measures, disability weights should be empirically assessed in South Africa and other LMICs, rather than based on regional data or data from different countries.

Even DALYs cannot fully capture the societal costs of mental disorders because they do not take into account the indirect costs, such as lost productivity of patients and carers, household resources spent caring for a sick family member, travel costs for hospital visits, or the negative impact on patients’ children who may not receive adequate attention and care. Indirect costs are particularly high for MNS disorders and must be examined alongside disease burden to fully illustrate their effects on patients, their families, and the broader society (77). Furthermore, most direct and indirect cost studies conducted in sub-Saharan Africa examine mental disorders broadly, with few studies differentiating the costs due to particular conditions and none specifically examining costs of neurological and substance use disorders. More disorder-specific cost data are needed to inform decisions about priority setting and investment.

Second, although use of traditional medicine for mental disorders is common in South Africa (50, 51), there are no data on the prevalence of use, motivations for use, costs, or effectiveness of these treatments. A better understanding of why people use traditional medicines and the health effects of a range of traditional practices would provide insight into beliefs about mental health, how traditional healing could complement or be integrated with conventional medicine, and whether any of these practices put patients at risk. Additionally, existing research on traditional treatments for other health conditions suggests that the costs for traditional medicine are high in South Africa, often as high as those for conventional medical care, and are typically borne by the poorest segment of the population (78), yet there is no cost data available on traditional interventions for MNS disorders. Complete, accurate data on traditional medicines and their costs would provide a more thorough picture of mental health care and could provide a basis for cost-effective, integrated interventions that operate in tandem with existing traditional practices.

Third, effective strategies for integrating mental health services into other parts of South Africa's health system must be designed and tested. Interventions to incorporate mental health into primary care and into care for people living with HIV have shown promise for use in LMICs (79–81), but must be tested in South Africa. Few interventions that integrate attention to MNS disorders into treatment programs for other chronic diseases (82) or blend conventional treatment of MNS disorders with traditional medicines have been implemented and tested in a LMIC. Before interventions can be implemented on a national scale, they must be tested in South Africa and the effectiveness data used to guide scale-up.

Fourth, in concert with evaluation of the effectiveness of interventions, there is a need for more data on cost-effectiveness and the economic impact of a range of interventions and intervention packages. At present, cost-effectiveness research primarily examines specific treatments, rather than care packages, such as coordinated treatment for patients with co-morbid conditions, prevention efforts integrated with primary health care, or cooperation with traditional healers. Future cost-effectiveness studies will need to examine a broader selection of integrated interventions.

National or provincial cost-effectiveness data may differ substantially from global or regional findings and could be important for bringing about changes in funding priorities (26, 73). For instance, regional cost-effectiveness data do not fully account for inefficiencies in South Africa's fragile health system, such as high absenteeism and unfilled posts. Furthermore, there is a need to examine the broader societal benefits, such as gains in productivity and employment and reduction in costs to other parts of the economy, for instance, policing and crime, child protection, or social work services. Cost-effectiveness data can inform choices on resource allocation, and information on economic gains will help with advocacy for mental health services. These types of economic data are particularly important given the context of South Africa's planned implementation of a national health insurance.

## Limitations

The analysis and findings in this review must be acknowledged in light of several limitations. A narrative overview was selected rather than a systematic review because there is little economic research related to MNS disorders in sub-Saharan Africa. As a result, there is great need for an introduction to the topic that challenges current thinking, and defines the future research agenda—appropriate goals for a narrative overview (52). Although the authors defined the section headings prior to conducting the search, they took precautions to ensure the presentation of findings was as unbiased as possible, confining their commentary and interpretation to the discussion section and did not generate their arguments until results were drafted. The grey literature was not searched, and the authors did not approach the Ministry of Health to get additional unpublished data. Finally, costing data was converted to a single currency, but was not adjusted to account for inflation to preserve the integrity of the original data.

## Conclusion

This narrative overview examines the epidemiological context of MNS disorders in South Africa and reviews what is known about their costs and the cost-effectiveness of their treatments. Existing data suggests that providing mental health services in the context of other health interventions and prevention efforts aimed at limiting alcohol consumption may be most cost-effective. Further research on the costs related to MNS disorders is greatly needed to develop an evidence base to support effective and efficient implementation and advocacy.

Building political will is critical for the implementation of more integrated models of mental health care. Economic data will be one key factor in making a persuasive case and assisting policymakers to make more informed choices about the importance of investment in mental health care and inclusion of mental health in the basket of options for the proposed national health insurance. While this review has put forward a set of potential priorities for researchers to address, further analysis must be conducted in tandem with conversations with policymakers able to introduce changes based on the findings.

**Main findings**

- South Africa faces a growing burden of mental, neurological, and substance use (MNS) disorders, which are often co-morbid with HIV and other chronic diseases. A considerable mental health treatment gap exists, with significant care shortages in rural areas.
- Indirect costs, primarily from foregone income due to MNS disorders, are substantial in sub- Saharan Africa.
- The most cost-effective treatment interventions in sub-Saharan Africa incorporate mental health care into community-based services. Taxation of alcohol is a “best buy” for prevention.

**Key messages for action**

- Four policy-relevant knowledge gaps are identified in South Africa:
  - Epidemiological and economic burdens of MNS disorders must be fully understood to inform spending decisions
  - More data on the use, costs, and effectiveness of traditional therapies for MNS disorders are necessary to develop interventions that combine traditional and biomedical care
  - Effective strategies for integrating mental health services into primary care must be designed and tested
  - Context specific data on the cost-effectiveness of integrated intervention models of cares is essential for advocacy and spending choices
- Economic data is critical for advocacy, to develop integrated models of mental health care and will inform choices between competing spending priorities.

## References

[1] Murray CJ, Vos T, Lozano R, Naghavi M, Flaxman AD, Michaud C (2013). Disability-adjusted life years (DALYs) for 291 diseases and injuries in 21 regions, 1990–2010: a systematic analysis for the Global Burden of Disease Study 2010. Lancet.

[2] Saxena S, Thornicroft G, Knapp M, Whiteford H (2007). Resources for mental health: scarcity, inequity, and inefficiency. Lancet.

[3] Mathers CD, Loncar D (2006). Projections of global mortality and burden of disease from 2002 to 2030. PLoS Med.

[4] Beyenburg S, Mitchell AJ, Schmidt D, Elger CE, Reuber M (2005). Anxiety in patients with epilepsy: systematic review and suggestions for clinical management. Epilepsy Behav.

[5] Mayosi BM, Lawn JE, van Niekerk A, Bradshaw D, Abdool Karim SS, Coovadia HM (2012). Health in South Africa: changes and challenges since 2009. Lancet.

[6] Prince M, Patel V, Saxena S, Maj M, Maselko J, Phillips MR (2007). No health without mental health. Lancet.

[7] Ramokgopa G (2012). A milestone for mental health in South Africa. Afr J Psychiatry.

[8] Summit PitNMH (2012). The Ekurhuleni declaration on mental health—2012. Afr J Psychiatry.

[9] DoHSA (2011). National health insurance in South Africa. http://us-cdn.creamermedia.co.za/assets/articles/attachments/34471_nhi.pdf.

[10] Tomlinson M, Lund C (2012). Why does mental health not get the attention it deserves. PLoS Med.

[11] Shiffman J, Smith S (2007). Generation of political priority for global health initiatives: a framework and case study of maternal mortality. Lancet.

[12] Drummond MF, Sculpher MJ, Torrence GW, O'Brien BJ, Stoddort GL (2005). Methods for the economic evaluation of health care programmes.

[13] (2006). Disease control priorities related to mental, neurological, developmental and substance abuse disorders. http://whqlibdoc.who.int/publications/2006/924156332x_eng.pdf.

[14] Clark DM (2011). Implementing NICE guidelines for the psychological treatment of depression and anxiety disorders: the IAPT experience. Int Rev Psychiatry.

[15] Collins PY, Insel TR, Chockalingam A, Daar A, Maddox YT (2013). Grand challenges in global mental health: integration in research, policy, and practice. PLoS Med.

[16] World Bank (2013). GINI index. http://data.worldbank.org/ondicators/si.pov.gini.

[17] Mayosi BM, Flisher AJ, Lalloo UG, Sitas F, Tollman SM, Bradshaw D (2009). The burden of non-communicable diseases in South Africa. Lancet.

[18] (2001). World health report 2001: mental health: new understanding, new hope.

[19] World Health Organization Mental health atlas: 2005. http://www.who.int/mental_health/evidence/mhatlas05/en/.

[20] Williams SL, Williams DR, Stein DJ, Seedat S, Jackson PB, Moomal H (2007). Multiple traumatic events and psychological distress: the South Africa stress and health study. J Trauma Stress.

[21] Stein DJ, Seedat S, Herman A, Moomal H, Heeringa SG, Kessler RC (2008). Lifetime prevalence of psychiatric disorders in South Africa. Br J Psychiatr.

[22] Kleintjes S, Flisher A, Fick M, Railoun A, Lund C, Molteno C (2006). The prevalence of mental disorders among children, adolescents and adults in the Western Cape, South Africa. Afr J Psychiatry.

[23] Havenaar JM, Geerlings MI, Vivian L, Collinson M, Robertson B (2008). Common mental health problems in historically disadvantaged urban and rural communities in South Africa: prevalence and risk factors. Soc Psychiatr Psychiatr Epidemiol.

[24] Herman AA, Stein DJ, Seedat S, Heeringa SG, Moomal H, Williams DR (2009). The South African Stress and Health (SASH) study: 12-month and lifetime prevalence of common mental disorders. S Afr Med J.

[25] Kessler R, Aguilar-Gaxiola S, Alonso J, Chatterji S, Lee S, Ormel J (2009). Special articles. The global burden of mental disorders: an update from the WHO World Mental Health (WMH) surveys. Epidemiol Psychiatr Sci.

[26] Gureje O, Chisholm D, Kola L, Lasebikan V, Saxena S (2007). Cost-effectiveness of an essential mental health intervention package in Nigeria. World Psychiatr.

[27] Kessler RC, Angermeyer M, Anthony JC, de Graaf R, Demyttenaere K, Gasquet I (2007). Lifetime prevalence and age-of-onset distributions of mental disorders in the World Health Organization's World Mental Health Survey Initiative. World Psychiatr.

[28] Lynskey MT, Strang J (2013). The global burden of drug use and mental disorders. Lancet.

[29] Baxter AJ, Patton G, Scott KM, Degenhardt L, Whiteford HA (2013). Global epidemiology of mental disorders: what are we missing?. PLoS One.

[30] Seedat S, Stein DJ, Jackson PB, Heeringa SG, Williams DR, Myer L (2009). Life stress and mental disorders in the South African stress and health study. S Afr Med J.

[31] Norman R, Bradshaw D, Schneider M, Pieterse D, Groenewald P (2006). Revised burden of disease estimates for the comparative risk factor assessment, South Africa 2000.

[32] Flisher AJ, Liang H, Laubscher R, Lombard CF (2004). Suicide trends in South Africa, 1968–90. Scand J Publ Health.

[33] Els C, Boshoff W, Scott C, Strydom W, Joubert G, Van der Ryst E (1999). Psychiatric co-morbidity in South African HIV/AIDS patients. S Afr Med J – Cape Town Medical Association of South Africa.

[34] Olley BO, Seedat S, Stein DJ (2006). Persistence of psychiatric disorders in a cohort of HIV/AIDS patients in South Africa: a 6-month follow-up study. J Psychosom Res.

[35] Myer L, Smit J, Roux LL, Parker S, Stein DJ, Seedat S (2008). Common mental disorders among HIV-infected individuals in South Africa: prevalence, predictors, and validation of brief psychiatric rating scales. AIDS Patient Care STDs.

[36] Freeman M, Nkomo N, Kafaar Z, Kelly K (2007). Factors associated with prevalence of mental disorder in people living with HIV/AIDS in South Africa. AIDS Care.

[37] Singh D, Berkman A, Bresnahan M (2009). Seroprevalence and HIV-associated factors among adults with severe mental illness: a vulnerable population. S Afr Med J.

[38] Cook JA, Cohen MH, Burke J, Grey D, Anastos K, Kirstein L (2002). Effects of depressive symptoms and mental health quality of life on use of highly active antiretroviral therapy among HIV-seropositive women. J Acquir Immune Defic Syndr.

[39] (2007). Epilepsy and HIV—a dangerous combination. Lancet Neurol.

[40] Lin EH, Korff MV (2008). Mental disorders among persons with diabetes—results from the World Mental Health Surveys. J Psychosom Res.

[41] James BO, Omoaregba JO, Eze G, Morakinyo O (2010). Depression among patients with diabetes mellitus in a Nigerian teaching hospital. S Afr J Psychiatr.

[42] Issa BA, Yussuf AD, Baiyewu O (2007). The association between psychiatric disorders and quality of life of patient with diabetes mellitus. Iranian J Psychiatr.

[43] Oladiji J, Akinbo S, Aina O, Aiyejusunle C (2009). Risk factors of post-stroke depression among stroke survivors in Lagos, Nigeria. Afr J Psychiatr.

[44] Nubukpo P, Clément J, Houinato D, Radji A, Grunitzky E, Avodé G (2004). Psychosocial issues in people with epilepsy in Togo and Benin (West Africa) II: quality of life measured using the QOLIE-31 scale. Epilepsy Behav.

[45] Mbewe EK, Uys LR, Nkwanyana NM, Birbeck GL (2013). A primary healthcare screening tool to identify depression and anxiety disorders among people with epilepsy in Zambia. Epilepsy Behav.

[46] Flisher AJ, Lund C, Funk M, Banda M, Bhana A, Doku V (2007). Mental health policy development and implementation in four African countries. J Health Psychol.

[47] World Health Organisation (2011). Mental health atlas 2011. http://www.who.int/mental_health/publications/mental_health_atlas_2011/en/.

[48] Lund C, Kleintjes S, Kakuma R, Flisher AJ (2010). Public sector mental health systems in South Africa: inter-provincial comparisons and policy implications. Soc Psychiatr Psychiatr Epidemiol.

[49] Petersen I, Lund C (2011). Mental health service delivery in South Africa from 2000 to 2010: one step forward, one step back. S Afr Med J.

[50] Sorsdahl K, Flisher A, Wilson Z, Stein D (2010). Explanatory models of mental disorders and treatment practices among traditional healers in Mpumulanga, South Africa. Afr J Psychiatry.

[51] Sorsdahl K, Stein DJ, Flisher AJ (2010). Traditional healer attitudes and beliefs regarding referral of the mentally ill to Western doctors in South Africa. Transcult Psychiatr.

[52] Green BN, Johnson CD, Adams A (2006). Writing narrative literature reviews for peer-reviewed journals: secrets of the trade. Journal of Chiropractic Medicine.

[53] South African Department of Health, DOHS SA (2012). Mental health policy framework for South Africa and strategic plan 2014–2020 (final draft) Pretoria.

[54] Hu TW (2006). Perspectives: an international review of the national cost estimates of mental illness, 1990–2003. J Ment Health Pol Econ.

[55] McLeod H, Rothberg A, Pels L, Eekhout S, Mubangizi DB, Fish T (2002). The costing of the proposed chronic disease list benefits in South African Medical Schemes in 2001. http://www.commerce.uct.ac.za/Research_Units/CARE/RESEARCH/Papers/ChronicDiseaseListReport.pdf.

[56] Truter I (2010). Prescribing of drugs for Alzheimer's disease: a South African database analysis. Int Psychogeriatr.

[57] Provinces spend 46% of combined capital budgets. http://www.sanews.gov.za/south-africa/provinces-spend-46-combined-capital-budgets.

[58] Petersen I, Lund C, Bhana A, Flisher AJ (2012). A task shifting approach to primary mental health care for adults in South Africa: human resource requirements and costs for rural settings. Health Pol Plann.

[59] Lund C, Boyce G, Flisher AJ, Kafaar Z, Dawes A (2009). Scaling up child and adolescent mental health services in South Africa: human resource requirements and costs. J Child Psychol Psychiatry.

[60] Gillis L, Koch A, Joyi M (1990). The value and cost-effectiveness of a home-visiting programme for psychiatric patients. S Afr Med J.

[61] Lund C, Myer L, Stein DJ, Williams DR, Flisher AJ (2013). Mental illness and lost income among adult South Africans. Soc Psychiatr Psychiatr Epidemiol.

[62] Esan OB, Kola L, Gureje O (2012). Mental disorders and earnings: results from the Nigerian National Survey of Mental Health and Well-being (NSMHW). J Ment Health Pol Econ.

[63] Kirigia J, Sambo L (2003). Cost of mental and behavioural disorders in Kenya. Ann Gen Psychiatr.

[64] Kessler RC, Andrews G, Colpe LJ, Hiripi E, Mroczek DK, Normand S-LT (2002). Short screening scales to monitor population prevalences and trends in non-specific psychological distress. Psychol Med.

[65] Canavan ME, Sipsma HL, Adhvaryu A, Ofori-Atta A, Jack H, Udry C (2013). Psychological distress in Ghana: associations with employment and lost productivity. Int J Ment Health Syst.

[66] Patel V, Araya R, Chatterjee S, Chisholm D, Cohen A, De Silva M (2007). Treatment and prevention of mental disorders in low-income and middle-income countries. Lancet.

[67] Chisholm D, Sanderson K, Ayuso-Mateos JL, Saxena S (2004). Reducing the global burden of depression Population-level analysis of intervention cost-effectiveness in 14 world regions. The British Journal of Psychiatry.

[68] Chisholm D (2005). Cost-effectiveness of first-line antiepileptic drug treatments in the developing world: a population-level analysis. Epilepsia.

[69] Chisholm D, van Ommeren M, Ayuso-Mateos JL, Saxena S (2005). Cost-effectiveness of clinical interventions for reducing the global burden of bipolar disorder. The British Journal of Psychiatry.

[70] Chisholm D, Rehm J, Van Ommeren M, Monteiro M (2004). Reducing the global burden of hazardous alcohol use: a comparative costeffectiveness analysis. J Stud Alcohol Drugs.

[71] Anderson P, Chisholm D, Fuhr DC (2009). Effectiveness and costeffectiveness of policies and programmes to reduce the harm caused by alcohol. Lancet.

[72] Chisholm D, Gureje O, Saldivia S, Villalón Calderón M, Wickremasinghe R, Mendis N (2008). Schizophrenia treatment in the developing world: an interregional and multinational cost-effectiveness analysis. Bull World Health Organ.

[73] Chisholm D, Saxena S (2012). Cost effectiveness of strategies to combat neuropsychiatric conditions in sub-Saharan Africa and South East Asia: mathematical modelling study. BMJ.

[74] Siskind D, Baingana F, Kim J (2008). Cost-effectiveness of group psychotherapy for depression in Uganda. J Ment Health Policy Econ.

[75] Choosing interventions that are cost effective. http://www.who.int/choice/costs/CER_thresholds/en/.

[76] Colton CW, Manderscheid RW (2006). Congruencies in increased mortality rates, years of potential life lost, and causes of death among public mental health clients in eight states. Prev Chronic Dis.

[77] Hyman SE (2010). The diagnosis of mental disorders: the problem of reification. Annu Rev Clin Psychol.

[78] Nxumalo N, Alaba O, Harris B, Chersich M, Goudge J (2011). Utilization of traditional healers in South Africa and costs to patients: findings from a national household survey. J Publ Health Pol.

[79] Blank MB, Hanrahan NP, Fishbein M, Wu ES, Tennille JA, Ten Have TR (2011). A randomized trial of a nursing intervention for HIV disease management among persons with serious mental illness. Psychiatr Serv.

[80] Crepaz N, Passin WF, Herbst JH, Rama SM, Malow RM, Purcell DW (2008). Meta-analysis of cognitive-behavioral interventions on HIV-positive persons’ mental health and immune functioning. Health Psychol.

[81] Kaaya S, Eustache E, Lapidos-Salaiz I, Musisi S, Psaros C, Wissow L (2013). Grand challenges: improving HIV treatment outcomes by integrating interventions for co-morbid mental illness. PLoS Med.

[82] Ngo VK, Rubinstein A, Ganju V, Kanellis P, Loza N, Rabadan-Diehl C (2013). Grand challenges: integrating mental health care into the non-communicable disease agenda. PLoS Med.

